# Strictly-posterior thoracotomy: A minimal-access approach for construction of the modified Blalock-Taussig shunt in West African children

**DOI:** 10.11604/pamj.2014.17.106.3791

**Published:** 2014-02-12

**Authors:** Frank Edwin, Baffoe Gyan, Innocent Adzamli, Mark Tettey, Kow Entsua-Mensah, Martin Tamatey, Lawrence Sereboe, Ernest Aniteye, Nana Akyaa-Yao

**Affiliations:** 1 National Cardiothoracic Center, Korle Bu Teaching Hospital, P O Box KB 846, Accra, Ghana

**Keywords:** Congenital heart disease, tetralogy of Fallot, modified Blalock-Taussig shunt, minimal-access surgery, thoracotomy, polytetrafluoroethylene

## Abstract

**Introduction:**

In resource-poor settings, the modified Blalock-Taussig shunt (MBTS) is often performed for symptomatic relief of Fallot's tetralogy. From September 2011, we adopted the strictly posterior thoracotomy (SPOT), a minimal-access technique for the MBTS and report the cosmetic advantages in this communication.

**Methods:**

We retrospectively analyzed the records of consecutive patients in whom the SPOT approach was used to construct the MBTS. Study end-points were early mortality, improvement in peripheral oxygenation, morbidity, and the cosmetic appeal.

**Results:**

Between September 2011 and January 2013, 15 males and 8 females, median age 4 years (1.3 - 17 years) and weight 13 kg (11 - 54 kg) underwent the MBTS through the SPOT approach. The polytetrafluoroethylene grafts used ranged from sizes 4 - 6mm (median 5mm). The median preoperative SpO2 was 74% (55% - 78%), increasing to a postoperative median value of 84% (80% - 92%). Shunts were right-sided in 22 patients and left-sided in one. There were no shunt failures. Hospital stay ranged from 7 - 10 days. There was one early death (4.3%), and two postoperative complications (re-exploration for bleeding and readmission for drainage of pleural effusion). The surgical scars had excellent cosmetic appeal: they ranged from 5-10 cm in length; all were entirely posterior and imperceptible to the patient.

**Conclusion:**

The SPOT approach represents a safe and cosmetically superior alternative to the standard posterolateral thoracotomy, the scar being imperceptible to the patient. The excellent cosmetic appeal and preservation of body image makes this approach particularly attractive in children and young adults.

## Introduction

In the current era, early primary repair is the preferred option in the management of tetralogy of Fallot with pulmonary stenosis. However, in resource-poor regions, this approach is often not feasible due to economic constraints relating to funding of open heart procedures[1]. Consequently, the modified Blalock-Taussig shunt (MBTS) is performed for symptomatic relief of Fallot's tetralogy in patients unable to afford the out-of-pocket expense for primary complete correction. We reported earlier that only 20% of the parents of children (less than 15 years old) requiring surgery for congenital heart disease in West Africa are able to finance the operation within 12months of diagnosis [2]. Thus by default, the MBTS becomes both the initial and destination therapy for a considerable number of late-presenting children with severe cyanosis. A second (contralateral) MBTS may indeed be performed on the same premise when the first shunt becomes inadequate with growth.

The original approach for the modified Blalock-Taussig shunt (MBTS) as described by de Leval and coworkers [3] was a posterolateral thoracotomy. In recent times, the median sternotomy has evolved to become the favored approach for the MBTS; subsequent complete repair then entails a repeat sternotomy. Although a repeat sternotomy accomplishes the staged repair with one incision, it is more time consuming and carries a 1.3% risk of reentry injury, though the mortality risk is not increased [4].

Our preferred approach for the MBTS has been the posterolateral thoracotomy. However, the aesthetic insult from posterolateral thoracotomy has been the basis of complaints from many parents/patients after an otherwise successful operation. Furthermore, the posterolateral thoracotomy has been associated with musculoskeletal deformities, especially in children with congenital heart defects [5] and the resulting scar affected the choice of clothing and/or caused embarrassment in 56% of patients in another study [6]. As the results of cardiac surgery continue to improve, the increasing importance of aesthetics has been emphasized. Previous investigators [7] have observed that the psychological burden of a full sternotomy should not be underestimated in children, teenagers, and young adults. The desire especially among the youth, for a ‘perfect‘ body image has contributed to the impetus for less-conspicuous thoracotomies in the repair of simple congenital heart lesions. On the bases of these concerns, we adopted a strictly posterior thoracotomy (SPOT) for the MBTS beginning from September 2011. To the best of our knowledge, this minimal-access approach has not been reported for the construction of the MBTS.

## Methods

**Figure 1:**
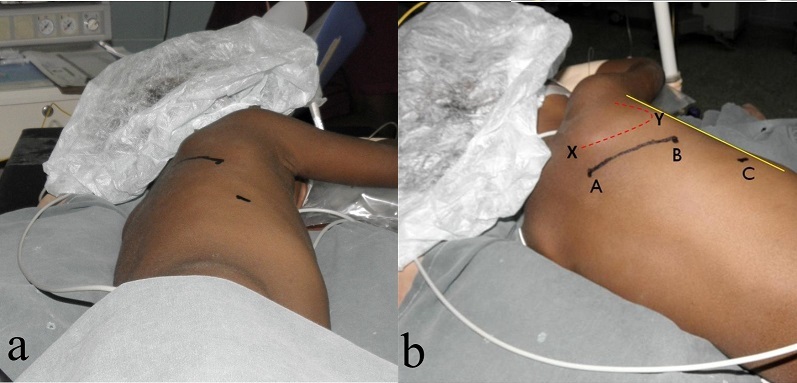
a) Black marker indicates proposed skin incision and chest drain site; b) The incision runs from A to B; C – chest drain site; yellow line – posterior axillary line; dashed line – outline of scapula (X – spine; Y – angle)

**Figure 2:**
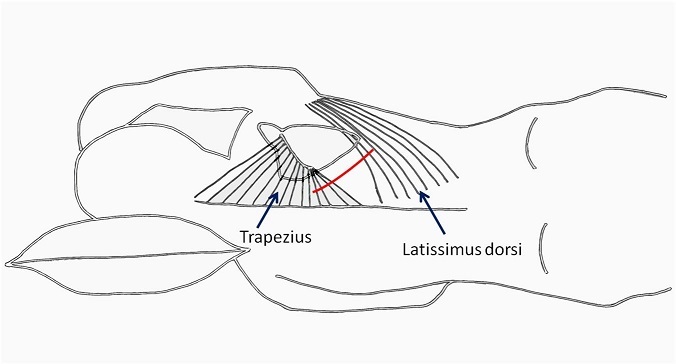
The trapezius and latissimus dorsi are divided (minimally) in the line of the incision (red line)

**Figure 3:**
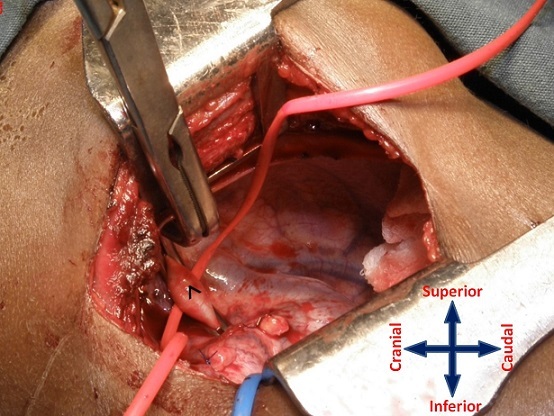
Exposure and control of the right subclavian artery (^ with red vessel loop) for arteriotomy and subsequent anastomosis

**Figure 4:**
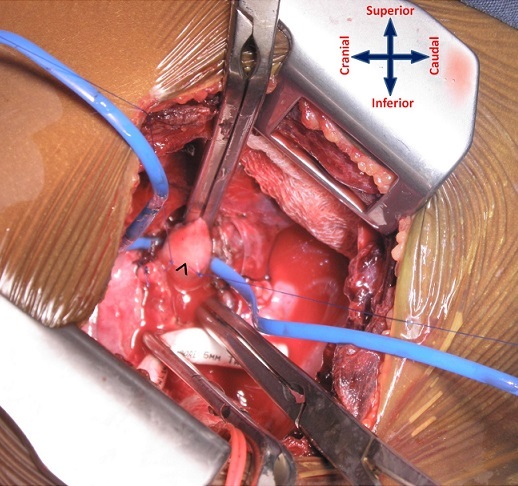
Exposure and control of the right pulmonary artery (^) in preparation for arteriotomy and anastomosis

From September 2011 to January 2013, 23 patients with cyanotic congenital heart disease and reduced pulmonary blood flow who underwent the MBTS through the SPOT approach were selected for retrospective analysis. Their case notes were reviewed to ascertain demographics and surgical outcome in terms of mortality, morbidity, and the cosmetic result. *Definitions* Strictly posterior thoracotomy: We defined the strictly posterior thoracotomy (SPOT) as a 6-8cm incision between the scapular spine and posterior axillary line, 2 cm parallel to the vertebral border of the scapula. *Early shunt failure*: The MBTS is deemed to have failed if complete occlusion is documented during the hospitalization period, or a second shunt was required during the same hospitalization on account of poor oxygenation (SpO2 < 80%).*Statistical Analysis* Statistical analysis was performed using statistical software on Microsoft Office Excel 2007. Results are expressed in percentages, mean ± standard deviation, and median with range. *Surgical technique* The MBTS was performed on the right side in 22 patients and on the left side in one. *Position*: For a right MBTS, the patient is placed in a left lateral decubitus position with a slight counter-clockwise tilt, keeping the elbow flexed and positioned on an arm rest (Figure 1). The position is maintained using pads beneath the head and chest. Additional support is obtained using straps across the hip in standard fashion. *Incision*: The incision is kept strictly posterior to the posterior axillary line. It begins from the level of the base of the scapular spine (T3 vertebra) and extends 6-8 cm towards the angle of the scapula, about 2cm parallel to the vertebral border of the laterally rotated scapula (Figure 1 b, line A-B). It is carried through the skin and subcutaneous tissues until the fasciae overlying the latissimus dorsi and trapezius muscles are exposed. The posterior border of the latissimus dorsi is divided 1cm across its width while sparing the serratus anterior; the inferior border of the trapezius is also divided for about 1cm across its width (Figure 2). The chest is entered through the 4th intercostal space. After opening the pleura, a retractor is positioned to enable gentle retraction of the ribs. The lung is retracted and held inferiorly using wet sponges instead of a malleable retractor; this reduces of the restricted operative field (Figure 3, Figure 4).

*Vessel exposure*: The subclavian artery is exposed after incising the overlying mediastinal pleura; the artery is dissected down to its origin from the innominate artery. Care must be taken not to injure the overlying ansa subclavia during the dissection of the subclavian a National Cardiothoracic Center, Korle Bu Teaching Hospital, P O Box KB 846, Accra-Ghanartery origin.

Prior to dissection of the right pulmonary artery, the azygos vein is divided to facilitate exposure of the pulmonary artery between the superior caval vein anteriorly and the right bronchus posteriorly. The peri-arterial sheath of the pulmonary artery is dissected to fully mobilize the vessel proximal to the origin of the upper lobe branch. Once isolated, the subclavian and pulmonary arteries are encircled with vessel loops and controlled.

*Anastomoses*: It is important to ascertain that unilateral lung perfusion is able to sustain adequate oxygenation when the pulmonary artery is clamped during the anastomosis; the pulmonary artery is test-clamped for a few minutes to evaluate this before proceeding with the vascular anastomoses. An appropriate size polytetrafluoroethylene (PTFE) graft is chosen based on the patient´s age, body weight, and size of the branch pulmonary arteries. The graft is tailored to fit the distance between the subclavian and pulmonary arteries, avoiding redundancy. Heparin is administered intravenously (100 units/kg) before applying the vascular clamps (Figure 3, Figure 4). The subclavian and then the pulmonary anastomoses are constructed end-to-side with the PTFE interposition graft using continuous 6/0 or 7/0 polypropylene suture. Just prior to completion of the pulmonary anastomosis, the vascular clamp is released and an intraoperative venesection is performed (with concurrent replacement using an appropriate volume of crystalloid), aiming to lower the hematocrit to about 50%.

**Figure 5:**
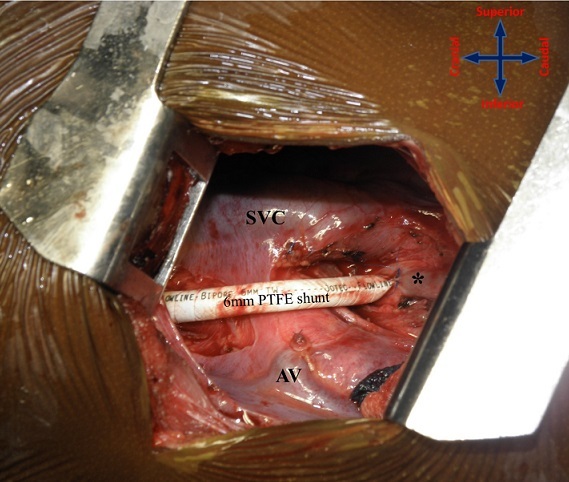
Completed 6mm PTFE shunt lying in straight trajectory without redundancy or kinks. AV – azygos vein (divided); SVC – superior vena cava; * – right pulmonary artery

A thrill is palpable over the graft when an adequately patent shunt has been constructed. A reduced diastolic pressure and improved peripheral oxygen saturation provide additional proof of shunt patency. The completed shunt should lie in a direct trajectory between the subclavian and pulmonary arteries without tension or redundancy (Figure 5).

*Chest drainage and closure*: The chest drain is placed in the 5th or 6th interspace in the posterior axillary line (Figure 2; point C). Pericostal sutures are used to close the intercostal space. The divided parts of the trapezius and latissimus dorsi muscles are reconstructed. The skin is closed using absorbable intradermal sutures.

*Postoperative Care*: Patients are monitored in the Intensive Care Unit after the procedure and transferred to general ward care when hemodynamically stable. Trans-thoracic echocardiogram is performed to confirm shunt patency prior to hospital discharge.

## Results

**Figure 6:**
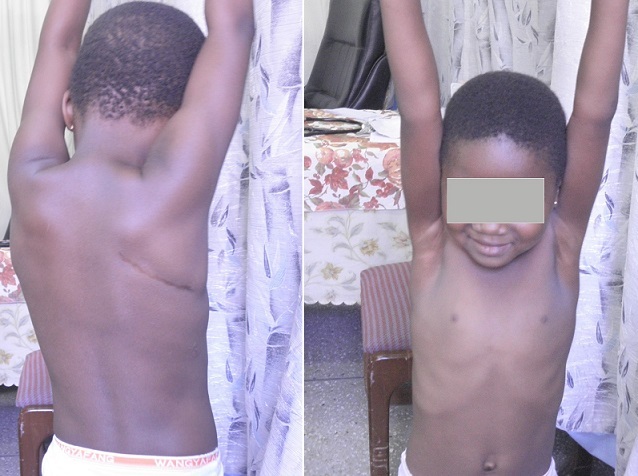
The scar from the SPOT incision is inperceptible to the patient

Twenty-three patients underwent the MBTS through the SPOT incision of length ranging from 5-10 cm on the basis of body size. All patients had symptomatic tetralogy of Fallot (21) or double-outlet right ventricle with restricted pulmonary blood flow (2). There were 15 males and 8 females, median age 4 years (1.3 - 17 years) and weight 13 kg (11 - 54 kg). The PTFE grafts used ranged from sizes 4 - 6mm (median 5mm). The median preoperative SpO2 was 74% (55% - 78%), increasing to a postoperative median value of 84% (80% - 92%). All patients were weaned from mechanical ventilation within 2 hours after the procedure and were discharged from the Intensive Care Unit the day after the procedure. Hospital stay ranged from 7 - 10 days; there were no shunt failures. The only mortality (4.3%) occurred in a 17 year old male who died suddenly on the 7th postoperative day from a suspected arrhythmia; the autopsy confirmed long-standing untreated Fallot's tetralogy but was otherwise unrevealing. Two (8.6%) important postoperative complications occurred: one re-exploration for bleeding (no surgical bleeding found), and one readmission for a serous pleural collection that required tube drainage 7 days after discharge. Three of the patients experienced delayed wound healing (extra 7 days) managed on out-patient basis. The cosmetic result was generally excellent (Figure 6).

## Discussion

As the safety of cardiac surgery has improved over the years, the cosmetic sequel of operations has gained increasing importance. The shift to a more cosmetically-appealing incision, however, warrants consideration only when surgical safety, optimal accomplishment of surgical goals, and avoidance of important postoperative complications are not compromised. The SPOT approach was therefore assessed in terms of the safety of the technique, adequacy of the surgical exposure, and the cosmetic result. *Safety of the technique* The technique's safety was considered in terms of shunt patency rates achieved, early mortality, and morbidity. Before the adoption of the SPOT approach in our institution, we had achieved a 92.4% success rate at a mortality of 3.8% and morbidity of 11.8% using the posterolateral thoracotomy in 264 patients undergoing the MBTS between 1992 and 2011 [8]. Other workers in similar settings reported a 77.9% success rate, 6.7% mortality rate, and 8.7% morbidity rate associated with the creation of the MBTS through a posterolateral thoracotomy [1]. Using the SPOT approach, the success rate has been 100% so far, with a mortality of 4.3% and morbidity of 8.6%, results comparable to that obtained using posterolateral thoracotomy.

*Surgical exposure* We found the exposure obtained using the SPOT approach to be adequate for the smooth performance of the MBTS. So far, conversion to the standard posterolateral thoracotomy has not been required. The instrumentation required is standard and not different from what would be used in the posterolateral approach. Procedural technique and surgical precision was not compromised by the minimal-access incision; and because the incision is an abbreviated version of the posterolateral thoracotomy, it can be easily extended laterally when deemed necessary for obtaining more liberal exposure.

The minimal-access incision, however, makes manipulation of surgical instruments more challenging (especially for the assistant surgeon) and renders a second assistant redundant. Yet, as we have shown, surgical outcomes are not compromised when the technique is performed by surgeons skilled in the standard techniques. The restricted exposure portends an additional learning curve and probably makes the SPOT approach unsuitable for trainees in their early careers.

*Cosmetic result* The SPOT approach yields a superior cosmetic result (Figure 6): the scar is imperceptible to the patient and does not cause psychological distress from daily confrontation in the dressing mirror. As a result, the perception of body image is preserved. This is an important advantage since much of the dissatisfaction associated with surgical scarring is a result of the patient's perception of disfigurement and the associated social stigmatization. The conspicuous midline sternotomy scar, for example, has been described as a lifelong reminder of a 'heart problem' not only to the patient but to his entourage as well. [7] The SPOT scar, lying completely outside the patient's field of vision (Figure 6), should not trigger such a label or sentiment. In an age where the perfect body image is almost idolized in the media and much of society, this advantage is expected to be highly valued among the young in whom body image perception contributes significantly to positive self-esteem.

Because of diminished surgical trauma, minimally-invasive approaches are generally held to lead to increased postoperative patient comfort and faster recovery. We did not objectively assess this in our study but subjectively, we noted less postoperative pain and earlier ambulation in the SPOT patients. As a corollary, delayed chest wall mal-development is considered unlikely due to the limited nature of the incision.

*Technical hints* Apart from good illumination with the surgical headlight and adequate magnification, the critical components for a smooth procedure using the SPOT approach are the following: • Positioning: lateral decubitus, slightly leaning forward (Figure 1); the pulmonary anastomosis is more difficult in the true lateral decubitus position. • The postero-superior limit of the incision should reach the level of the base of the scapular spine to facilitate the subclavian anastomosis. • Wet sponges for caudal retraction of the lung to minimize clutter of the operative field. The caudal lung traction should keep the mobilized pulmonary artery in an infero-lateral (rather than true lateral) position; the pulmonary anastomosis is much easier in this position. *The role of the MBTS in resource-poor settings* Currently, early primary correction of tetralogy of Fallot is the preferred management option. Early primary repair is often not feasible in West Africa due to late presentation, economic and logistic constraints. [1–2] The initial surgical option may thus be restricted to a palliative shunt in a child presenting late with cyanosis. Palliative shunts are often performed with the understanding that the shunt may eventually become destination therapy. From our experience, 67% of tetralogy patients successfully palliated did not return for total repair largely on economic grounds[2]. For the minority returning later for complete repair, the SPOT approach is still advantageous in preserving a virgin territory for sternotomy with an inconspicuous primary scar.

*Thoracotomy versus sternotomy for MBTS* The thoracotomy approach has the advantage that subsequent total correction can be performed through virgin territory without the adhesions occasioned by a previous sternotomy. However, the additional scar on the chest after total correction through sternotomy is cosmetically unappealing. In the current era, more than 90% of congenital heart surgeons in the developed world prefer sternotomy for the MBTS [9]. Sternotomy is technically, a less demanding approach for the MBTS, providing greater control of the innominate and pulmonary arteries. The sternotomy approach, used initially for the creation of central PTFE aortopulmonary shunts in smaller infants demonstrated the benefits of shunting without distortion of the peripheral pulmonary arteries [10]. The shift from the traditional posterolateral thoracotomy towards sternotomy gained momentum as other workers [11] reported that the MBTS performed through a thoracotomy approach has 4 times the risk for shunt failure as compared to a sternotomy approach. More recent workers have not demonstrated the superiority of shunt patency reported with the sternotomy approach; rather there are indications that the median sternotomy approach for MBTS may have a higher morbidity in terms of longer ventilation times, longer duration of inotropic support, intensive care, and hospital stay [12]. Some have even suggested that the sternal approach for MBTS construction is an independent risk factor for in-hospital death [9], although it is conceivable that sternotomy is merely a surrogate for a higher risk patient or procedure. Possibly, the outcomes are similar for either approach. As pertains in much of the developing world, when staged repair of Fallot's tetralogy is unavoidable, the SPOT approach to the MBTS provides the advantageous virgin sternotomy for subsequent total repair together with an improved cosmetic appeal of an inconspicuous thoracotomy. *Study limitations* The relatively small number of patients implies that the full repercussions of this minimal-access technique are yet to be known. Additionally, long term results are not available to compare with those of the standard approaches. We adopted this approach on the basis of prior experience that the MBTS may become destination therapy for a large number of our patients. Subjectively, we have presumed that should subsequent complete repair become financially possible for the patient, a single sternotomy scar for a staged repair is the cosmetic equivalent of an imperceptible SPOT scar plus a median sternotomy. We have not objectively assessed our presumption, which is an issue deserving further study.

## Conclusion

The MBTS can be safely constructed using the minimally invasive SPOT approach, a cosmetically superior alternative to the standard posterolateral thoracotomy. The excellent cosmetic result makes this approach particularly attractive in children and young adults in the developing world in whom the MBTS may become destination therapy on socio-economic grounds.
